# Unlock the drivers of early ANC visits among pregnant women in Kasulu town council, Tanzania: an institutional cross-sectional study

**DOI:** 10.1186/s12978-025-02162-3

**Published:** 2025-10-07

**Authors:** Winfrida Benedicto Lyoba, Chakupewa Joseph Mpambije, Joyce Donald Mwakatoga

**Affiliations:** 1 Department of Global Health and Bio-Medical Science, The Nelson Mandela African Institution of Science and Technology, P.O Box 447, Arusha, Tanzania; 2 Department of Health System and Impact Evaluation and Policy, Ifakara Health Institute, P.O Box78373, Dar-es-Salaam, Tanzania; 3 Department of History, Political Science and Development Studies, Mkwawa University College of Education, University of Dar es Salaam, P.O Box 2512, Iringa, Tanzania; 4 Department of Agricultural Extension and Community Development, Sokoine University of Agriculture, P.O Box 3002, Morogoro, Tanzania

**Keywords:** Tanzania, Pregnant women, Antenatal care visit, First trimester

## Abstract

**Background:**

Maternal and child mortality remains a global public health challenge. Thus, countries, including Tanzania, have adopted different cost-effective models, especially antenatal care (ANC) to improve maternal and child health (MCH). Despite the early timing of ANC visits having a great implication for ensuring improved MCH services, Tanzania has disproportionately experienced late ANC visits among pregnant women. This has entailed conducting an institutional-based study in Western Tanzania, Kasulu Town Council (KTC) to ascertain whether demographic socio-economic and maternal characteristics imply the persistence of late ANC visits using robust methods.

**Methods:**

An institutional cross-sectional study design was conducted in KTC, Kigoma Region using an embedded mixed-method approach from March-April 2020. Quantitative data was collected from 320 women with children aged 0–6 months attending postnatal services. A total of 40 participants were involved in the qualitative study through in-depth interviews with healthcare providers and focus group discussions held with pregnant women and women with children aged 0–6 months. Descriptive statistics and multivariate binary logistic regression were used to determine the characteristics associated with the timing of ANC visits among pregnant women. Furthermore, thematic analysis was used to generate themes triangulated with quantitative results.

**Results:**

Findings revealed that 32.2% of pregnant women attended ANC visits in the first trimester. Early ANC was associated with maternal age (AOR = 1.839, 95% Cl: 1.023, 3.303), being accompanied by a partner (AOR = 2.165, 95% Cl: 1.256, 3.733), and awareness of the danger signs (AOR = 2.079, 95% Cl: 1.172, 3.687) and parity (AOR = 2.164, 95% Cl: 1.091, 4.291). Little association was noted in the knowledge of ANC timing (AOR = 0.564, 95% Cl: 0.320, 994) and household income (AOR = 0.529, 95% Cl: 0.281, 0.995). Qualitative data indicated that low rate of early ANC initiation was attributed to a lack of support from partners and accompanied to ANC visits, insufficient knowledge of the timing of early ANC visits, and socio-cultural beliefs.

**Conclusion:**

Results confirmed that early ANC visit in KTC is low. Revealed associated factors act as a bridge to improve maternal and newborn health and contribute to achieving Sustainable Development Goal no 3, which targets maternal mortality of less than 70 deaths per 100,000 live births and neonatal mortality of 12 per 1000 live births by 2030. Proposed integrated interventions can potentially ensure that women, regardless of pregnancy status, are encouraged to receive early ANC utilisation during the first trimester to receive antenatal care before delivery to improve maternal and newborn health.

**Supplementary Information:**

The online version contains supplementary material available at 10.1186/s12978-025-02162-3.

## Introduction

Globally, maternal and child mortality has significantly dropped by one-third over the past 20 years​ [1]. In the same period, global maternal deaths were 223 per 100,000 live births, with almost 95% of all maternal deaths occurring in low and lower-income countries (LMICs) and 253,000 (87%) deaths in Sub-Saharan Africa (SSA) and Southern Asia [1]. According to the WHO Report 2020, the SSA region had the highest annual maternal mortality rate of 202,000 (70%), while 47,000(17%) deaths occurred in Southern Asia [1]. Maternal and newborn deaths revealed to occur among women who do not receive adequate Antenatal Care (ANC) services and delayed obstetric emergency care during pregnancy [2]. Various global efforts have been proposed and implemented to improve maternal and child health care. For instance, WHO has recommended essential ANC for positive pregnancy experiences to provide pregnant women with comprehensive care. This requires a pregnant woman to make eight ANC visits in developing countries: at least one ANC visit in the first trimester, two in the second trimester, and five in the third trimester [3, 4]. Timely ANC visits and adequate utilisation of ANC services from skilled health care providers (HCPs) is the aspirant and effective strategy to improve maternal health and reduce neonatal mortality [5, 6].

Wondemagegn et al., confirmed that higher maternal mortality in SSA is inaccessible and delayed ANC visit initiation [7]. About 25% of maternal deaths occur during ANC visits, primarily owing to pre-eclampsia, eclampsia, and antepartum haemorrhage, which can be addressed during ANC [8] and reduces risks of maternal mortality (MMR) [9]. The recent trend of maternal and child mortality from Africa revealed that MMR stood at 189 per 100,000 live births, under-five mortality at 52 per 1000, and neonatal mortality at 22/1000 live births [10]. In Nigeria, MMR was 512/100,000 live births, under-five mortality rate (U5MR), and neonatal mortality132/1000 and 39/1000 live births respectively [11]. Ethiopia portrayed MMR with 312/100,000 live births, 47/1000 for under-five, and 34/1000 for infants [12]. In Zambia, MMR stood at 252 per 100,000 live births, the under-five mortality rate was 61 per 1000, and neonatal 27/1000 live births [13], while Sierra Leone has the highest MMR of 1360 per 100,000 live births [14]. Given the unsatisfactory trend of MMR in SSA, Tanzania has adopted the ANC visit recommended by WHO to reduce maternal, neonatal, and infant mortality [15]. This led to increased institutional delivery to 81% from 51% within the last five years [16]. Furthermore, women WHO made the first ANC visits in the first trimester and four ANC visits increased from 22% to 34% and from 51% to 65% in 2016 and 2022, respectively [16]; however, early ANC visits in the first trimester remain relatively stumpy. In Tanzania, MMR dropped to 104 per 100,000 live births in 2022 compared to 556 per 100,000 live births in 2016 [16]. Similarly, U5MR dropped from 67/1000 to 43/1000 and from 43/1000 to 33/1000 for infants in 2016 and 2022 respectively [16]. This improvement owes to diverse interventions implemented to improve MCH services alongside ANC improvements [17].

Concerning MMR rates, various regions of SSA are strongly characterised by low utilisation of ANC services [3]. Studies in SSA revealed that women initiate their first ANC visit late which is against the WHO recommendations [18, 19]. For example, adherence to the recommendations was below 45% and less than 25% in developing countries and SSA respectively [20]. Studies conducted in some specific countries revealed different proportions of initiation of ANC visits during the first trimester. Specifically, the proportions were 18% in Nigeria [11], 20.1% in Uganda [21], 21.7% in Ethiopia [2], and 37% in Zambia [13]. In Tanzania, the proportion of pregnant women WHO attended ANC during the first trimester increased from 22% in 2016 to 34% in 2022, while those WHO attended at least four visits increased from 51% in 2016 to 65% in 2022 [16]. In the Kigoma Region, women WHO attended ANC more than four visits accounted for 60.8%, and those WHO made ANC visits in the first trimester comprised 40.6% while 20.5% made zero ANC visits during pregnancy [16]. The ANC platform provides prevention and curative services to both pregnant women and their fetus. To recognise these benefits, Ambaye, Regasa, & Hailiye, 2023 reported that prevention interventions made during ANC visits including counselling, healthy eating education, and physical exercises could prevent excessive weight gain during pregnancy detection [5]. Moreover, during ANC visits, mothers can test for HIV status, detect hemoglobin levels and diabetes, identify fetal abnormalities, and create awareness of danger signs [22].

The empirical evidence from Ethiopia and Nepal demonstrated how the low ANC uptake is associated with maternal age [23, 28], education [23, 24], occupation [25], and marital status ​ [26, 27]. On the one hand, income [28], lack of knowledge about early ANC visits ​ [26, 29], unplanned pregnancy [30, 31], number of children and spouses accompanying policy [29] were associated with late ANC visits. Other factors include the husband’s education [32] and religion [27]. Correspondingly, women who lived nearest health facilities were more likely to attend ANC visits during the first trimester [26] and parity [22]. On the contrary, women who live in rural communities were less likely to utilise ANC in the first trimester [26]. Further, traditional gender roles, fear of shame and stigma, cultural beliefs about pregnancy, rude language of health personnel, and shortage of HCPs were associated with late ANC [29]. Despite the above factors, little is known, especially in the Western part of Tanzania, especially the Kigoma Region, about the associated demographic, socio-economic, and maternal characteristics hindering pregnant women from initiating ANC visits in the first trimester.

Tanzania has ratified the global agenda to achieve goal number three of the Sustainable Development Goals (SDG) by 2030: to reduce global MMR to less than 70 deaths per 100,000 live births and U5MR to 25 live births [33]. While Tanzania has made a great stride in reducing MMR and U5MR, the reduction of neonatal mortality is still unacceptably high at 24 per 1000 live births [16]. Moreover, the assurance of achieving the SDGs target is still uncertain, thus requiring continued adoption of effective strategies to improve ANC, especially in remote areas like Kigoma Region. However, understanding the factors limiting initiation of ANC during the first trimester using mixed methods has a potential role in providing information to intervention planners, policymakers, HCPs, and stakeholders to create a road map towards the timing of first ANC initiation, increased number of ANC visits and utilisation of ANC services during pregnancy. Therefore, the study provides recent information about demographics, socio-economic, maternal, and health system factors essential for enhancing ANC visits in Kasulu Town Council (KTC), Kigoma Region.

## Methods

### Study design, setting, and population

This study was carried out from March to April 2020 in KTC using embedded mixed methods, both quantitative and qualitative, with a cross-sectional design. The KTC is one of the eight councils in the Kigoma Region, located in the north-western part of Tanzania, bordering Burundi, Uganda, Congo, and Rwanda [34]. Table 1 presents, as indicated, the traits of the research milieu. Regarding the population portrayed according to the 2022 census, KTC had a population of 238,321 (126154 females and 112167 males). The KTC comprises two (2) Divisions and 15 Wards [34, 35].

**Table 1:** Key characteristics of the study setting

Parameter	Data
Population	238,321
Wards	15
Public and private hospitals	2
Public health center	3
Public and private dispensaries	30
Health workers available	169 (48%)
Shortage of health workers	353 (52%)
Maternal mortality rate	199/100,000
Infant mortality rate	20/1000
Neonatal mortality rate	15/1000
Institutional delivery	94%
ANC attendance	79.5%
Current stunting situation	3.6

Source: URT 2022; CCHP, 2022/23–25; URT2021; THDS, 2022; FHR 2024

The KTC has 47,663 households comprising a population 238,321 and 35 health facilities; more than 50% are public​ [35, 36]. KTC has 35 health facilities, two hospitals, one public hospital, and another faith-based hospital. In addition, 3 public health centres and 30 dispensaries were found in KTC, of which 12 are owned by the private sector [36, 37]. The MMR stood at199/100,000 live births in the area, slightly below the national average [34]. The nutritional status of U5MR is not impressive, with 203 severely stunted cases, 366 wasted cases, 587 under-weights, and 1089 born with low birth weight [34]. The number of HCPs was 169, with a shortage of 353, equal to 52% [34], which threatens effective health service delivery. Generally, these characteristics link individual behaviour to broader health system and demographic challenges and make more meaningful and actionable on how to influence ANC attendance rather than looking at an individual level. Mortality and prevalence of stunting can be used to examine late ANC visits as contributing factors. In addition, the number of health facilities and healthcare providers determines the quality, accessibility and availability of ANC services, directly influencing the timely initiation of ANC visits. Similarly, reporting ANC attendance rate helps to make a comparison with the national (65%) and helps to assess whether the study findings are representative of the broader trend [16].

### Study variables

#### Dependent and independent variables

The dependent variable was the timing of the first visit to ANC services based on the WHO recommendation that the first ANC visits should be made in the first trimester (< 12 weeks) [5]. Gestational age was used to establish the timing of the ANC visit, and it was calculated from the Last Normal Menstrual Period (LNMP). A woman who attends ANC visit < 12 weeks (3 months) was considered to have initiated ANC in the first trimester. On the other hand, ultrasound (6–12 weeks) and Fundal Height Measurement are used to confirm gestational age, especially LNMP, which is uncertain. The timing of ANC visits was categorised into two segments: one (1) is the initiation of ANC visit in the first trimester (< 12 weeks), and zero (0) is initiation of ANC visit > 12 weeks. The independent variables captured include demographic, socio-economic, and maternal characteristics such as age, education, religion, occupation, marital status, information, timing of ANC, number of ANC, and number of children. All these characteristics have an influence on the timing of ANC visits, frequency and quality of ANC visits in KTC. For instance, younger women may have less knowledge about the importance of ANC or face social stigma, leading to fewer visits while older mothers are more likely to seek care due to increased awareness associated with pregnancy and vice versa. Additionally, information on partner accompaniment, distance to the health facility, nutritional counseling, time spent at the facilities, availability of ANC components like iron-folic acid supplementation, anti-worms and anti-malaria were captured using women’s satisfaction, perception and their lived experience to determine the quality of the services provided at the ANC facility. The findings were used to recommend on the improvement of timely ANC attendance and maternal and child health outcomes.

#### Sample and sampling technique

**Fig. 1:**
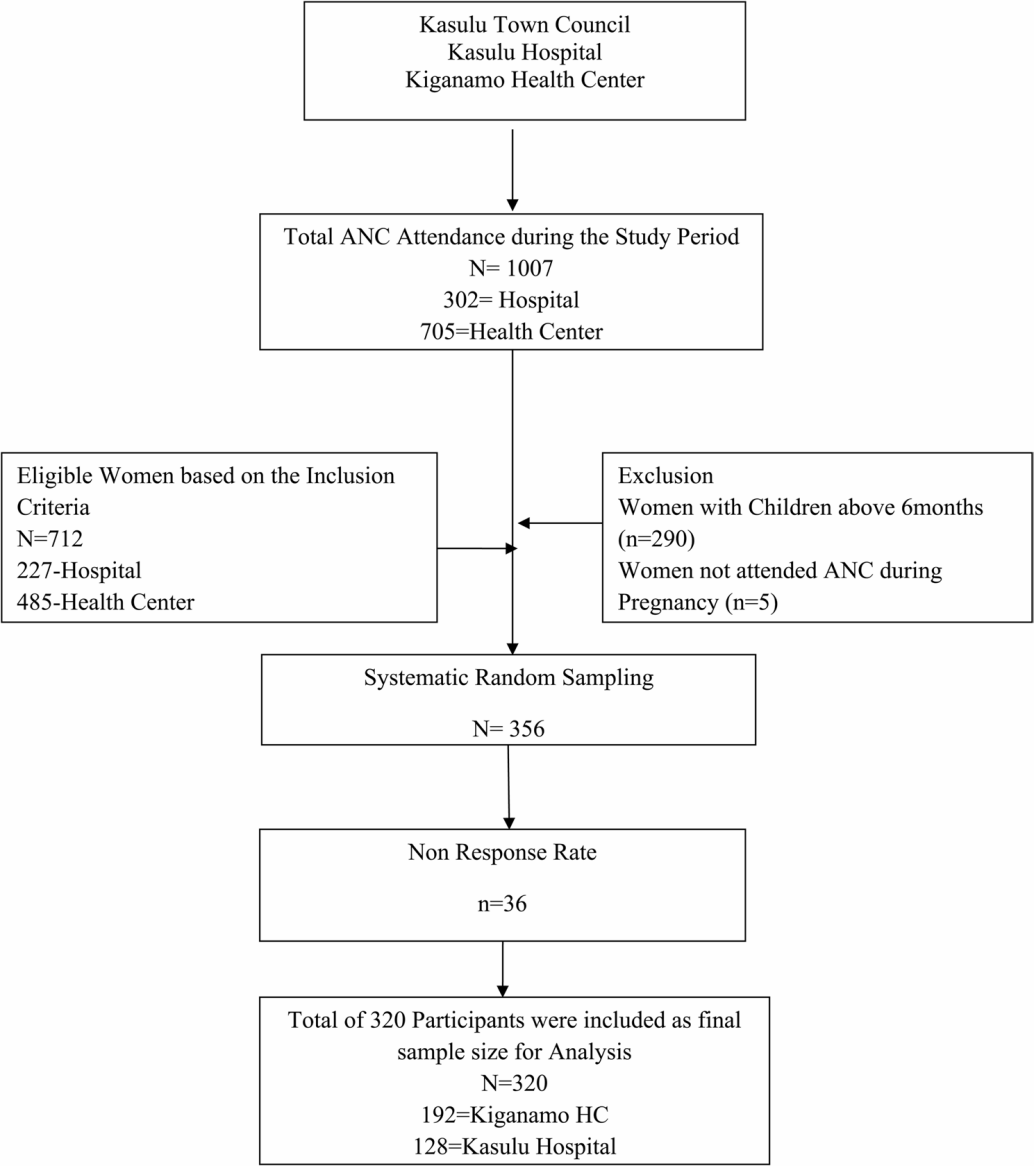
Participant flow chart diagram

The study covered two facilities in KTC: Kasulu Hospital and Kiganamo Health Centre. These facilities were selected based on statistical representativeness with a broader population receiving services in high caseloads to iµprove statistical power and reduce saµpling bias. In addition, these facilities were selected to ensure the availability of the data, validity and generalisation. Further, quantitative data was obtained from 320 women with children aged 0–6 months attending ANC during the study period. Women with children aged 0–6 were recruited to minimize recall bias and ensure participant could accurately recall their ANC attendance history. This approach allowed the study to capture data on completed ANC visits and understand the WHOle trajectory of ANC utilisation from the first day of visit to delivery to improve the reliability of the reported timing of ANC visits. A quantitative method was employed to determine the proportional proportion of pregnant women who initiated ANC visits below 12 weeks of pregnancy and determinants associated with ANC initiation including age, education, income, health systems and obstetric factors for proper decision-making. Figure 1 shows the participant flow chart diagram on how sampling of participants were conducted.

The information on their socio-economic, demographic, and maternal characteristics associated with the timing of ANC during pregnancy were assessed at a 95% confidence level (Zα/2 = 1.96) by considering the national proportion of timing of ANC (34%)​ [16], 80% desired power (Bα) and 10% of the estimated non-responsive rate. The population proportion formula was used to calculate the sample size and 10% of non-respondents, incomplete responses and dropouts were considered to maximise the sample size to account for the categorical data analysis. The 10% was derived from standard research practices and deemed moderate and reasonable without overestimating face-to-face interaction to maintain the validity and reliability of the findings. To ensure the community proportion gives a good estimate at facility level, selected facilities were based on statistical representativeness with a broader population receiving services with high caseloads to iµprove statistical power and reduce saµpling bias, ensuring data availability, validity and generalisation.

Qualitative facilities were selected using purposive saµpling, diversity, key inforµants, and expert availability to obtain rich, in-depth insights related to the research topic and understand the context and lived experience. In-depth interviews (IDIs) and focus group discussions (FGDs) were conducted to complement quantitative data. The FGD and IDI methods were used to explore women’s experiences, perceptions, barriers, and perspectives regarding ANC initiation from both HCPs and women. Cultural beliefs about ANC attendance, maternal health, family, community involvement and health systems were also explored to understand community knowledge. Both FGDs and IDIs involved 40 participants who were recruited purposefully. The sum of four (4) FGDs involved 34 women participants with an average of 7–11 participants per group and six (6) Health Care Providers (HCPs) from the reproductive care health (RCH) department were involved for IDI. These HCPs were recruited due to their experience in caring for pregnant women, lactating women, and the under-five children. Study tools were administered in Kiswahili and later translated into English for accuracy and consistency.

#### Inclusion and exclusion criteria

Criteria for the selection of health facilities included considering services provided, including ANC Postnatal Care (PNC) services and all clinical services (Outpatients and Inpatients). Similarly, these facilities were selected because they serve the whole population in KTC, have a high caseload to iµprove statistical power, reduce saµpling bias and for better representation. Women of reproductive age (15–49 years) with children aged 0–6 months attending ANC and PNC services were selected on the day of the study. The sampling frame comprises a systematic list of participants from the target population attending PNC. Further, the HCPs and Nutrition Officers working at RCH and ANC sections were selected purposively and involved in data collection if they were present during the study. Generally, women with children above 6 months, those who did not consent and were unable to speak/hear, or had maternal disorders were excluded from the study.

### Data collection procedure and tools

#### Quantitative method

A structured questionnaire was employed to collect quantitative data about demographic, socio-economic, and maternal characteristics, including age, education, religion, occupation, marital status, information, timing of ANC, and number of ANC visits. Additional characteristics were the number of children, partner accompaniment, distance to the health facility, and perceived quality of the services provided at the ANC. Before data collection, WBL conducted training for enumerators to ensure a common understanding of the data collection tools and accuracy, integrity, and validity. The training conducted among enumerators covered topics on how to capture information and keep confidential information before data collection.

#### Qualitative method

Regarding qualitative data, the social scientists, CJM and JDM, with experience in collecting qualitative data, trained the research assistants to moderate, take notes, and capture reactions during interviews and FGDs. The tools were translated into Kiswahili and back to English for accuracy and consistency. Before the actual data collection, a pre-test was conducted to ensure the reliability of the collected data. In fact, during the actual data collection, qualitative data was captured through FGDs and IDIs, whereas quantitative data was collected using a questionnaire. The entire team administering questionnaires, FGDs, IDIs, and filled forms and notebooks were collected from the authors and checked before data analysis.

### Data management and analysis

Before analysis, quantitative data was checked, coded, and entered into the SPSS software version 22, with a statistical significance of p-value < 0.05. In addition, the quality of the collected data was checked using descriptive analysis. The dependent variable was the timing of ANC at the first visit (< 12 weeks vs. > 12 weeks) and independent variables include age, education, knowledge on the number of ANC, parity, occupation, education, gravidity, knowledge on the danger signs, accompanied by partner etc.) Then, the binary logistic regression model was employed after running a multicollinearity diagnosis to establish the characteristics associated with the first ANC. Indeed, initiation of ANC care in the first trimester (< 12 weeks (3 months), was estimated using the Adjusted Odd Ratio (AOR). The AOR quantifies the likelihood of early ANC initiation based on influencing factors including access to health care services, maternal education, awareness, income etc. A p-value < 0.05 and 95% Cl indicate that factors associated have statistical significance and reliability with early ANC initiation and do not happen by chance. Gestational age was used to establish timing of ANC visit which was calculated from the Last Normal Menstrual Period (LNMP). A woman WHO attends ANC before 12 weeks (3 months) was considered to have initiated ANC in the first trimester. On the other hand, ultrasound (6–12 weeks) and Fundal Height Measurement were used when turning to the second trimester to confirm gestational age, especially when LNMP is uncertain.

All audio-recorded information was transcribed for qualitative data analysis to generate important themes for meaningful information. Simultaneous listening and reading of the transcript were done to ensure the accuracy of the information. Considering thematic analysis for qualitative findings, NVIVO software was employed and raised themes were triangulated with the quantitative report. In addition, knowledge of the timing of ANC visits was measured based on understanding the timing of the first ANC visit in < 12 weeks of pregnancy, and at least mention two benefits of timing of ANC visit. Participants who responded to two questions correctly were considered to understand the timing of ANC visits. Knowledge of danger signs was measured using the eight signs, and women who mentioned at least half of them were considered to understand them.

### Ethical consideration

The Ifakara Health Institute (IHI) Review Board approved the study on 09 February 2020 (IHI/IRB/No: 9-2020). An introduction letter from the Department of Research and Training of Ifakara Health Institute was also provided. The purpose of the study was explained to all study participants, and written informed consent forms were signed before the interviews. Verbal consent was enquired before conducting FGDs. Before conducting the study, the participants who were unable to read and write were asked to sign using a fingerprint and ensure the confidentiality of information.

## Results

Findings involving 320 participants from Kiganamo Health Centre and Kasulu Hospital were presented. A total of 192 accounts to 60% (60%) of participants attended at Kiganamo Health Centre, and remain 128 were from Kasulu Hospital. The demographic, socio-economic, and maternal characteristics of women with children aged 0–6 months were reported.

### Demographic characteristics

Table 2 illustrates demographic characteristics such as mother’s age, education, marital status, husband’s age, and education. Regarding age, 44% of the women participants were 15–24 years. This proportion was high considering the sensitivity of the adolescent group, reported to face more barriers to initiating ANC visits than mature women. Over 50% (172) of the respondents had completed primary education, which is still low with implications in decision-making on their health. In contrast, 142 (45%) of the respondents aged between 25 and 34 years and 172 (53.8%) had completed secondary education and above respectively. However, age and education may imply differences in decision-making regarding health service-related matters. In addition, married women account for 285 (89.1%) of the respondents.

**Table 2:** Demographic characteristics of participants in KTC, (*N* = 320)

Variable	Characteristics	*n*	%
Mother’s age (years)	15–24 years 25–34 years 35–49 years	140	43.8
		121	37.8
		59	18.4
Partner’s age	15–24 years	62	19.4
	25–34 years	142	44.9
	35+ years	116	36.7
Mothers education	No formal education Completed primary Completed secondary +	69	21.6
		172	53.8
		79	24.7
Partner’s education	No formal education Completed Primary Completed secondary +	69 172	21.5 24.7
		79	53.8
Marital status	Single	35	10.9
	Married	285	89.1

Source: Field data, 2020

### Socio-economic characteristics

The occupation of mothers and their partners has significant implications for the first ANC visits. About 232 (72.5%) of women were not employed in the formal system, and 88(27.5%) were employed in the private sector through self-employment either small farming activities or informal sectors. Contrary, about 140 husbands or partners equal to 43.8% were not employed. Regarding health-seeking behaviour, faith in various religions was an important factor to consider among participants. The findings unveiled that protestants and followers of other religions were 167 (52%), followed by Roman Catholics with 90 participants (28%) and 63 (20%) were Muslims as illustrated in Table 3.

**Table 3:** Socio-economic characteristics of participants in KTC (*N* = 320)

Variable	Characteristics	*n*	%
Mothers Occupation	Not employed	232	72.5
	Employed	88	27.5
Partner’s Occupation	Not employed	140	43.8
	Employed	180	56.2
Mothers Religion	Catholic Protestant Christian Muslim	90	28.1
		167	52.2
		63	19.7
Number of meals	Twice and Below Thrice +	174	54.4
		146	45.6
Household income Television ownership	Below 210,000 (Poor) 210,000–400,000(Average) 410000 + (High Yes No	237	74.1
		44	13.8
		39 98 222	12.2 30.6 69.4

Source: Field data, 2020

Regarding the number of meals consumed in a day about 174 (54.4%) households took their meals twice or less as an indication of the nutrition status in this community. In this community, most households were regarded as poor and cannot afford to cover the costs of basic needs based on the household income history. According to Table 3 above, about 237 (74.1%) had income below TZS, which was 210,000 (USD 84) monthly. On the other hand, ownership of communication devices like television (TV) has implications for education and behaviour change, however, 98 equal to 30.6% alone, owned TVs. Therefore, the majority of the community members would have limited information when disseminated through television.

### Timing of ANC visits and characteristics of participants

The timing of ANC visits was considered perfect if a woman attended the first ANC visit within the first trimester considering the quality of services provided. Women’s knowledge on the services received during pregnancy including counseling, nutritional advice, anaemia prevention, time spent to the facilities, availability of ANC components like iron-folic acid supplementation, anti-worms, HIV testing and Hb level, were captured using women’s satisfaction, and perception and their lived experience. Findings were used to recommend on the improvement of timely ANC attendance and maternal and child health outcomes. In the future, standalone study to assess quality of services and how is aligned with WHO recommendations. Gestational age was used to establish timing of ANC visits which was calculated from the Last Normal Menstrual Period (LNMP) and verified using registry book. A woman WHO attends ANC before 12 weeks (3 months) was considered to have initiated ANC in the first trimester. On the other hand, ultrasound (6–12 weeks) and Fundal Height Measurement when turn to second trimester to confirm gestational age especially LNMP is uncertain.

**Fig. 2:**
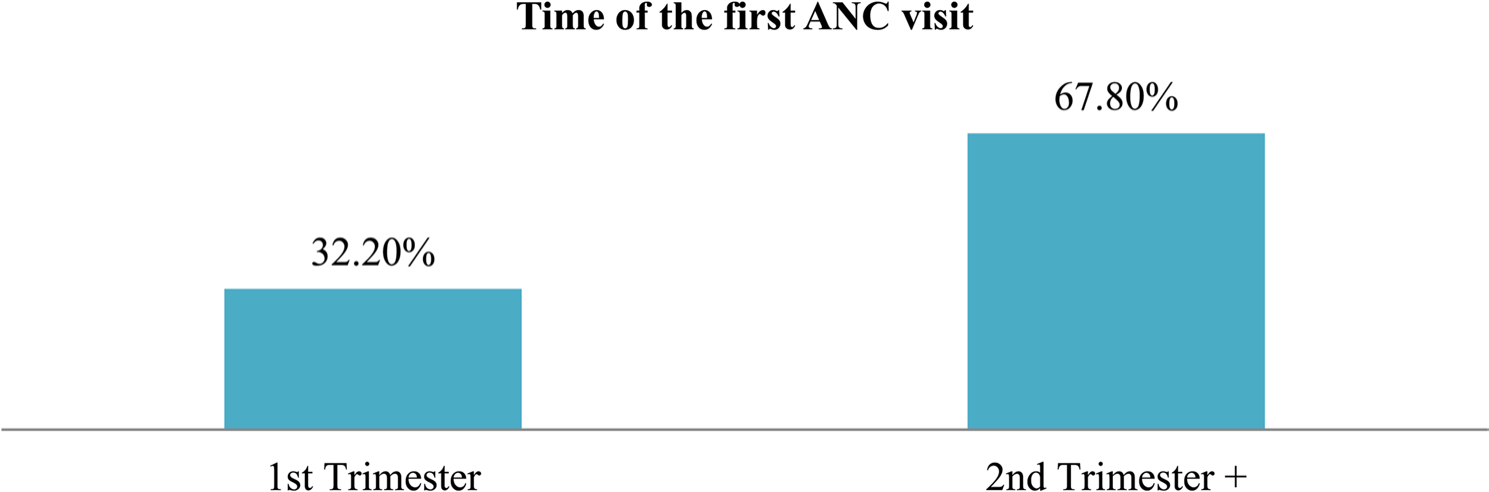
Timing of ANC visits

In this study, more than 60% of the participants initiated the first ANC visit in the second and third trimesters. Only 32% appeared for the first time in the first trimester. This is low proportional compared to the current national average of 34% in 2022, but high compared to 22% in 2016. Demographic attributes like social, economic, and maternal characteristics were associated with the timing of ANC visits. In addition, socio-belief, lack of knowledge about ANC visits, number of children, and lack of partner’s support were associated with late ANC visits. Figure 2 depicts the timing of ANC visits. The FGDs with pregnant women were conducted to capture their first-hand experiences, beliefs and barriers to timely ANC initiation. To complement FGDs, HCPs were interviewed because were interacting with diverse range of pregnant women in routine services and have a broader understanding of systemic challenges, health-seeking behaviour and patterns influencing early ANC visits which might not be fully captured through FGDs alone. This mixed perspective strengthen findings and improved the contextual understanding of ANC initiation drivers in KTC.

With regard to IDIs with HCPs, women appeared for ANC in the second and third trimesters. During key informant interview, HCP revealed that:

*“…In our community pregnant women attend ANC late*,* more women appear for ANC in the first time when the pregnancy is over five months”* (IDI: HCP, C-1).

Further, HCP disclosed that women who initiated ANC visits very late remain a challenge in the area; however, they provide them with education and explain the importance of early ANC visits using various methods, including a male involvement program. To emphasise this, one HCP said:

*“…most women start ANC visits within 5–6 months; others appear for the first time with 8 months of pregnancy*,* just to get a delivery slot”* (IDI: HCP, C-2).

Generally, the majority of women initiate ANC visits late in the second and third trimesters, with nonconstructive implications for maternal and child health. These include pregnancy complications during pregnancy, delivery, and post-delivery period. Intensive strategies are required to improve early ANC visits during pregnancy.

#### Awareness of the number of recommended ANC visits

The ANC visit has the potential to improve maternal and newborn health; however, most respondents were unaware of the importance of an adequate number of ANC visits to ensure safe delivery. Of 320 respondents, 272 (85%) were unaware of the recommended number of ANC visits during pregnancy. In regard to ANC visits, 62.2% of the respondents attended at least four visits. Respondents do not have a clear understanding of the eight recommended ANC visits. For example, some said it four times, and others said it five and six times. During FGDs, one said:

*“……I think any number of ANC visits is acceptable*,* whatever the pregnant woman can afford because of barriers differences”* (FGD: women who had children B-2).

According to WHO, the number of ANC visits a woman is encouraged to make eight visits to which women thought this number of visits was limiting for early ANC visits, as stated below.

“*Women complain about the number of ANC visits; they feel like the number of visits is a barrier to early ANC*,* as women in our area are disappointed with the number of ANC visits. They go to ANC so often but they can try to avoid it many times. Alternatively*,* women will return for an ANC visit late or visits early just and disappear for a duration of three complete months earlier than the subsequent visit”* (IDI: HCP, C-3).

Women expressed discomfort in attending ANC visits earlier and tended to avoid the frequency of eight ANC visits. They also cited that they were not aware of the recommended number of ANC visits and the problem of spending too much time simply going for ANC visits.

#### Parity

This corresponds to parity and determinants related to ANC visits. In this study, women with para 1–3 were 221 (69%) while those with para four (4) and above were 99 (31%). Parity affects decision-making with regard to the initiation of ANC visits. The majority of primigravida women tend to initiate ANC visits early when their partners or family members accompany them. Further details are explained in Table 4.

**Table 4:** Timing of ANC visits and characteristics of participants in KTC, (*N* = 320)

Variables	Frequency (*n*)	Percent (%)
Partner’s Age		
15–24 Years	62	19.4
25–34 Years	142	44.9
35- Above Years	116	36.7
Partner’s Education		
No formal education	32	10
Completed Primary	169	58.8
Completed Secondary+	119	37.2
Partner’s Occupation		
Employed	180	56.2
Not Employed	140	43.8
Parity		
1–3	221	69.1
4+	99	30.9
Number of ANC Visits		
Below four visits	121	37.8
Four and above visits	199	62.2
Number of children		
1–3 Children	221	69.1
4 + Children	99	30.9
Accompanied by husband/partner at ANC		
Yes	134	41.9
No	186	58.1
Knowledge about Timing for Early Visit		
Yes	115	35.9
No	205	64.1
Informed about Danger Signs		
Yes	212	66.2
No	108	33.8
Perceived Health Services		
Satisfactory	223	69.7
Unsatisfactory	97	30.3

Source: Field data, 2020

Parity is defined as the number of times a woman has given birth with a gestational age of 24 weeks or more including live births and stillbirths.

#### Accompanying partners

Partners’ support has implications for early or late initiation of ANC visits. The study found that of 320 women, those accompanied by their partners for the first ANC were 186 (58%). The same was revealed in the qualitative findings that the rate of male accompanying their partners was low. The issue of prevention of HIV transmission from mothers to children (PMCTC) was raised during FGDs. In Tanzania, it is mandatory for both partners to test for HIV/AIDS during the first ANC visits men who are not ready to know their HIV status tend to deny accompanying their partners. This guideline made pregnant women initiate ANC visits outside of the prescribed first trimester.

*“In my opinion*,* involving male partners when their wives visit for their first ANC is great. Nevertheless*,* the way it is implemented has far-reaching implications on when ANC visits should ideally begin. Let me give an example from my own experience and to be honest*,* I cannot find a reason why these processes should hurt me after all*,* I am married. After all*,* barriers related to HIV testing during pregnancy. I waited till my husband came back and initiated first ANC visits during my eighth months of this pregnancy*,* just imagine’’* (FGD: Women with children, B-3).

In addition, married women were not ready to face embarrassment for going for the first ANC visit without their male partners. The majority of women were ready to wait no matter how long it would take until their partners were ready to accompany them at ANC visits, especially in the first trimester. Cementing on such views, a woman during FGDs attested that:

“*One of the challenges we have in our society is that men do not accompany their partners to ANC. While partners are often uncomfortable to accompany their wives for the first ANC visit*,* women delay initiating ANC earlier”* (FGD: Women with children, B-4).

If a man is not ready to accompany his partner to the first ANC visit, women tend to wait but not ready to face discomfort from interrogation by HCPs. They are normally directed to seek approval from the village office, which warrants them with ANC services in the absence of their partners, which is also not comfortable. All these inconveniences make pregnant women delay their first ANC visits until they fulfill this conditions. This is an experience of a woman with her partner during FGD:

*“Men have negative views about ANC visits*,* but allow me to tell you the truth. For this pregnancy*,* I asked my husband to accompany me for the ANC visits and he said he would not follow me because it is not him who carrying a pregnancy. I was so depressed indeed. Even if you convince them*,* I really don’t know how they can be persuaded to come to ANC willingly”* (FGD: Pregnant Women-A3).

In this regard, males perceive that ANC visits are designed for women alone, and this perception continues to hinder the government’s efforts to recommend early ANC visits. Their resistance increases chances for maternal and child deaths that would otherwise be avoided. Pregnant adolescents are the most affected group, as they become pregnant out of socially unacceptable ways and the father is normally not exposed. This makes difficult for them to even go to the village chair and get a warrant to access ANC services. Thus, it becomes more difficult for them and therefore exacerbates more delays.

#### Knowledge about the timing of early ANC visits

The timing of the initiation of an ANC visit is crucial for maternal and newborn health. In this study, the proportion of women with proper knowledge about the timing of ANC visits was low at 36% (115). In regard to the knowledge revealed during FGDs with pregnant women when responding to the question on the proper time for starting ANC visits. One pregnant woman asserted that.

*“I am not sure when to begin antenatal care. However*,* the women around say it is after three to five months of pregnancy and others say the right time is just one to two weeks into the pregnancy. What I believe is that the first ANC visit should be done any time after realising that you an expecting mother’’* (FGD: Pregnant Women, A-1).

Similar respondents asked about the benefits of initiating ANC visits early. Most of them were unclear about the benefits of initiating ANC visits in the first trimester. One pregnant woman has something to say on the matter:

*“…. I don’t know*,* this is my first pregnancy*,* I would like to hear more about any advantage that can be gained if a person makes an early ANC visit”* (FGD: Pregnant Women A-2).

While the data collected across all sources depicted low knowledge among pregnant women on the benefit of initiating ANC within the first trimester, it is clear that limited intervention strategies at the community level and work overload among HCPs have attributed to low health-seeking behaviour among pregnant women.

#### Danger signs identification

A woman undergoes profound changes in their bodies after conception including experiencing diverse danger signs which if not managed early, may lead to maternal deaths. On this basis, a study sought to establish whether respondents could identify such signs. The study developed a list of danger signs for the respondents to identify them including severe virginal bleeding, convulsion, and severe headache with blurred vision, severe abdominal pain, and fatigue. On the other hand, listed danger signs were fast or difficulty breathing, reduced fetal movement, fever, and swelling of the fingers, face, and legs. Table 5 shows respondents’ capacity to identify danger signs women encounter during pregnancy.

**Table 5:** Knowledge on danger signs among participants in KTC, (*N* = 320)

Danger Signs	Frequency (*n*)	Percent (%)
Yes	212	66
No	108	44
Total	**320**	**100**

Source: Field data, 2020

Proper understanding of the danger signs during pregnancy is a good indicator of improving the health of the pregnant woman and their child. A woman who managed to identify at least five danger signs out of nine was considered to be informed by HCPs or other health promotion platforms, including radio, television, and family members. As shown in Table 5, only 212 (66%) of women managed to identify danger signs that women may encounter during pregnancy. A better understanding of danger signs is crucial during the planning of interventions to reduce maternal and child mortality. Despite the proportion of women informed about the danger signs, more efforts are needed to promote early utilisation of ANC services.

#### Perceived health services and timing of ANC

The majority of participants were positive about services provided during ANC visits and about 70% (234) were optimistic. This is notwithstanding the existing shortage of HCPs, which would have increased their burden. Similar findings echoed during FGDs, which hinted that the few available HCPs worked beyond their limit to ensure that ANC and additional health services were properly delivered. During FGD, study participants shared their experiences and perceptions of ANC services offered at the facilities.

*“…. I know from my own experience that nurses help us whatever late we are. Actually*,* nurses rushed into my delivery bed to attend me on my first pregnancy. But they would provide me instructions that helped me survive”.* (FGD: Women with Children (B-5).

A similar statement was provided by one HCP, who attested how they work hard to ensure that all those who appear for the ANC are attended, as quoted:

*“This facility is placed in a busy area so we get many clients at the RCH department and because of this we have made everything possible to meet their needs. This is despite them not meeting the diverse ANC guidelines by coming late*,* we give them all services including regular check-ups while pregnant”* (IDI: HCP, C-1).

Overall, women perceived the effectiveness of ANC services as an imperative tool in motivating them to seek ANC services. Pregnant women are assured that even when they delay seeking ANC services, they are still attended by HCPs from the facilities. Actually, services stipulated during ANC visits greatly benefit pregnant women and newborns if pregnant women adhere to ANC visits thoroughly.

### Demographic, socio-economic, and maternal characteristics associated with timing of ANC visits

Table 6 illustrates the factors associated with the timing of ANC visits during the first trimester as a potential period to reduce the number of maternal deaths and other negative outcomes. Regarding, the proportion of women who attended ANC visit in the first trimester during pregnancy was low. Reasons for delays were less motivation from their husbands and partners, cultural beliefs, knowledge of early ANC visits, and maternal age. The model used to assess the characteristics associated with the timing of ANC visits during pregnancy was significant at *P* < 0.05 with an adjusted R of 0.199 from Nagelkerke R square. The model was the best fit to predict the associated characteristics by 99.7%. A binary logistic regression model was used to detect the associated characteristics with ANC attendance, including maternal age, accompany of the husband or partner, and parity.

**Table 6:** Demographic, socio-economic, and maternal characteristics of participants associated with timing of ANC visits in KTC, (*N* = 320)

Variable Characteristics	Time of the First Visits (Months)		COR (Cl at 95%)	AOR (Cl at 95%)
(*n* = 320)	0–3 Months n (%)	4 + Months n (%)		
Mother Age				
15–24 Years	60 (58.25)	80 (36.87)	2.390 (1.484, 3.858)	1.839 (1.023, 3.303) *
25 + Years	43 (41.75)	137 (63.13)	1	1
Income				
Below 200,000 (Poor)	70 (67.97)	178 (82.03)	0.465 (0.271, 0.797)	0.529 (0.281, 0.995) *
210,000–400,000+ (High)	33 (15.53)	39 (17.97)	1	1
Knowledge about timing for ANC visits				
Yes	30 (29.13)	85 (39.17)	0.638 (0.385, 1.057)	0.564 (0.320, 994) *
No	73(70.87)	132 (60.83)	1	1
Food Security (no. of meals)				
Twice and Below	68 (66.02)	107 (49.31)	1.997 (1.227, 3.250)	1.493 (0.832–2.679)
Thrice +	35 (33.98)	110 (50.69)	1	1
Accompanied by Husband/Partner for ANC				
Yes	49 (47.57)	67 (30.88)	2.032 (1.254, 3.290)	2.165 (1.256, 3.733) *
No	54 (52.43)	150 (69.12)	1	1
Health Facilities attended				
KHC	58 (56.31)	134 (61.75)	0.798 (0.496, 1.285)	0.621 (0.351, 1.099)
KDH	45 (43.69)	83 (38.25)	1	1
Ownership of Television				
Yes	35 (33.98)	63 (29.03)	1.258 (0.761, 2.079)	0.789 (0.423, 1.474)
No	68 (66.02)	154 (70.97)	1	1
Awareness of Danger Signs				
Aware	79 (76.70)	133 (61.29)	2.079 (1.221, 3.540)	2.079 (1.172, 3.687) *
Not Aware	24 (23.30)	84 (38.71)	1	1
Parity				
1–3	84(81.55)	129 (59.45)	3.016 (1.711, 5.316)	2.164 (1.091, 4.291) *
4+	19 (18.45)	88 (40.55)	1	1

Source: Field data, 2020

*COR *Crude Odds Ratio, *AOR * Adjusted Odds Ratio, *Cl * Confidence interval

^*^statistically significant; 1 = reference; Hosmer and Leweshow Test = 99.7%. Therefore, the model was fitted to predict the characteristics associated with the timing of ANC visits

Indeed, early initiation of ANC among pregnant women attended at public facilities in Kasulu TC was significantly associated with six variables. Therefore, factors with high AOR should be considered when designing interventions to improve the timing of the utilisation of ANC in the future.

## Discussion

The study presented determinants associated with adhering to WHO recommendation that all pregnant women should make their first ANC visit in the first trimester [22, 39]. Results found that those initiating their ANC visit during the first trimester were only 32% however, this proportion was low compared to the national average of 35% [16]. Similar studies conducted in Ethiopia and Cameroon found that the timing of ANC visits in the first trimester was 30.6% and 36.5% respectively​ [40, 41]. On the other hand, the proportion of timing of ANC visits was high in Peru (52.9%) and Nepal (70%)​ [28, 42]. The studies found out certain aspects such as maternal age, male partner’s company, parity, household income, and awareness of the time to start the first ANC visit are associated with ANC attendance during the first trimester. As per authors from Ethiopia and Cameroon the observed differences could be associated with better health knowledge, cultural beliefs, partner support, economic status, and educational levels of pregnant women [40, 43].

The timing of ANC visits in the first trimester was associated with maternal age. For instance, younger women aged 24 and below were two (2) times more likely to delay attending ANC in the first trimester than older women. This is consistent with studies conducted in Tanzania from Tanzania in 2020 [44] and Manyeh et al. [41] in Ethiopia found that young women delayed ANC services. This is partly because young women fear to be judged and being mistreated or the fear to be expelled from schools and families, which makes them extend the time for the first ANC visit [45]. In Ghana, older women were more likely to attend ANC within the first trimester compared to young women [41]. On the contrary, findings were reported in Babor Zone Ethiopia and Uganda, which revealed that older women tended to attend their first ANC visits late​ [46, 47].

Early ANC attendance among pregnant women was associated with the knowledge of anaemia among mothers, experience in previous pregnancies, and perceived quality of health care services. In fact, knowledge among women in Ethiopia and India was reported to have a clear association with the timing of ANC visits [18, 23]. In Ethiopia, women who were advised before ANC visit were more likely to attend ANC early in the first trimester ​ [23, 30, 40]. Furthermore, women who were aware of the right time to start an ANC visit were more likely to attend the first visit early [31]. Knowledge differs across contexts because of the information provided, education sessions, and other programmes initiated to create awareness of ANC guidelines, including the number of ANC visits, the timing of ANC visits, and management of barriers such as fear of attending ANC visits [29].

In fact, the husband or partner has a role to play to ensure early ANC visits in the first trimester. Empirical evidence strongly revealed that women accompanied by partners increase the rate of ANC uptake within the first trimester. Similar findings were reported in Bhutan and Pakistani that women who received support from partners and family members were likely to attend ANC early [38, 48]. In Malawi and Ethiopia women who were not accompanied by their husbands or partners were less likely to attend the first ANC in the first trimester [39, 43]. Several reasons include couple relationship, knowledge of the husband about the importance of early ANC visits, male willingness, and the time spent at the facility. These were among the determinants of male partner support during the first ANC visit [27].

Alongside male participation, early ANC visit initiation lies in the quest for parity, which may enhance early ANC visits. However, a clear association between parity and early initiation of ANC services was unlocked. Many resource-constrained countries have persistently shown a correlation between parity and attendance of ANC, especially in the first trimester. For example, in Guinea and Pakistan, women with zero to three parties were more likely to appear for the first ANC visit in the first trimester than those with multi-parity​ [38]. This was revealed through the Ethiopian Health Demographic Survey of 2019, where multi-parity was associated with late ANC visits compared to primigravida [24]. In addition, according to findings from Pakistan, women who were multigravida with a high number of live births started ANC visits later than primigravida [38]. On the contrary, findings from Tanzania found that para one and above were more closely associated with late ANC visits ​ [44]. The inconsistency may be explained by implementing various interventions in diverse settings, women’s experiences from previous pregnancies, perceptions about ANC, and the existing socio-cultural factors [38, 48].

In regards to economic status, evidence has portrayed a relationship between household economic status and the ANC visit within the first trimester [29, 41]. While the study portrayed how pregnant women from poor households delayed initiating ANC within the first trimester [6]. Another finding from Nepal and Nigeria revealed that women from low-resourced countries appeared late at ANC whereas pregnant women from wealthy families attended ANC relatively early [28, 49]. However, a study in Ghana found that women from poor families were more likely to have timely ANC visits in the first trimester [41]. The quest for costs to cover health-related services, access to transport, and new dressings affects the early visit to ANC [29, 50]. Effects of economic status are more prominent in male-dominated communities because of their power to control household resources which deters women from starting early visits to ANC because they need to wait for their partner’s approval [51]. Additionally, poor economic status continues to threaten women even in situations where they experienced danger signs; they cannot make a quick decision to attend ANC for themselves [30].

## Conclusions

This study, conducted in Kasulu Hospital and Kiganamo health centre in KTC, revealed a low number of ANC visits during the first semester, of which only 32.2% successfully made it. The timing of the ANC visit was attributed to maternal age, parity, partner’s company, and awareness of danger signs. The findings from this study potentially suggest for undertaking tailored interventions geared to influence the early ANC covering the studied facilities and the entire KTC that exhibit similar features of late ANC visits. The proposed interventions will identify and address factors hindering pregnant women’s attendance to ANC in KTC. Such interventions are expected to create awareness about the potential benefits of early ANC visits, potential danger signs, and the promotion of male involvement. They will also improve health services and support to improve maternal and newborn health before, during, and after pregnancies. Interventions will also focus on expanding the scope of ANC services by providing ANC-related education before women become pregnant. It will be quite substantial for proposed interventions to also include male involvement aspects aimed to convince men to accompany and support their pregnant partners during the first ANC visit within the first trimester of pregnancy. Moreover, effective engagement of stakeholders to strengthen health system supporting the development of effective interventions to improve early ANC initiation is also preferred.

## Supplementary Information

Supplementary Material 1.

## Data Availability

No datasets were generated or analysed during the current study.

## Abbreviations

ANC: Antenatal Care
Cl: Confidential Interval
AOR: Adjusted Odds Ratio
FGD: Focus Group Discussion
IDI: In-depth Interview
HIV: Human Immunodeficiency Virus
VEO: Village Executive Officer
KTC: Kasulu Town Council
PMCTC: Prevention of Mothers to Child Transmission
(HCPs): Health Care Provider
TDHS: Tanzania Demographic Health Survey
