# Supplementary material for: Unlock the drivers of early ANC visits among pregnant women in Kasulu town council, Tanzania: an institutional cross-sectional study
Source: Reprod Health. 2025 Oct 7;22:187. doi: 10.1186/s12978-025-02162-3 (PMC12502604; doi:10.1186/s12978-025-02162-3)
Supplement: Supplementary file 1 — Supplementary Material 1. [file 12978_2025_2162_MOESM1_ESM.docx]

**Plain English Summary**

Antenatal care (ANC) is service provided to pregnant women to improve health of mother and fetus. According to World Health Organization (WHO), women are required to attend first ANC visit early with 12 weeks of pregnant, however, Tanzania timely ANC visit remain a challenge. This study was conducted to identify the factors for early initiation of ANC in Kasulu District in Kigoma Region. Data were collected from 320 women with children aged 0-6 months attending postnatal services. This was done through face to face interview using structured questionnaire. Also, four focus group discussion with 34 pregnant and women with children under 6 months was conducted. It was found that few pregnant women (32%) attend ANC during the first trimester. And the determinant for early antenatal visit is caused by maternal age and being accompanied by male partner. On other hand little association was noted in the knowledge of ANC timing and household income. In conclusion, early ANC visit among pregnant women was low compared to nation prevalence of 34%. When developing strategies that encourage early ANC visit, it is crucial to focus on integrated interventions that consider diverse socio-demographic characteristics that are embedded within the household and community level. .
